# Inhibition of Bitter Taste from Oral Tenofovir Alafenamide

**DOI:** 10.1124/molpharm.120.000071

**Published:** 2021-05-01

**Authors:** Erik Schwiebert, Yi Wang, Ranhui Xi, Katarzyna Choma, John Streiff, Linda J. Flammer, Natasha Rivers, Mehmet Hakan Ozdener, Robert F. Margolskee, Carol M. Christensen, Nancy E. Rawson, Peihua Jiang, Paul A.S. Breslin

**Affiliations:** Discovery Biomed, Birmingham, Alabama (E.S., J.S.); Monell Chemical Senses Center, Philadelphia, Pennsylvania (Y.W., R.X., K.C., L.J.F., N.R., M.H.O., R.F.M., C.M.C., N.E.R., P.J., P.A.S.B.); and Department of Nutritional Sciences, Rutgers University, New Brunswick, New Jersey (P.A.S.B.)Discovery Biomed, Birmingham, Alabama (E.S., J.S.); Monell Chemical Senses Center, Philadelphia, Pennsylvania (Y.W., R.X., K.C., L.J.F., N.R., M.H.O., R.F.M., C.M.C., N.E.R., P.J., P.A.S.B.); and Department of Nutritional Sciences, Rutgers University, New Brunswick, New Jersey (P.A.S.B.)

## Abstract

Children have difficulty swallowing capsules. Yet, when presented with liquid formulations, children often reject oral medications due to their intense bitterness. Presently, effective strategies to identify methods, reagents, and tools to block bitterness remain elusive. For a specific bitter-tasting drug, identification of the responsible bitter receptors and discovery of antagonists for those receptors can provide a method to block perceived bitterness. We have identified a compound (6-methylflavone) that can block responses to an intensely bitter-tasting anti–human immunodeficiency virus (HIV) drug, tenofovir alafenamide (TAF), using a primary human taste bud epithelial cell culture as a screening platform. Specifically, TAS2R39 and TAS2R1 are the main type 2 taste receptors responding to TAF observed via heterologously expressing specific TAS2R receptors into HEK293 cells. In this assay, 6-methylflavone blocked the responses of TAS2R39 to TAF. In human sensory testing, 8 of 16 subjects showed reduction in perceived bitterness of TAF after pretreating (or “prerinsing”) with 6-methylflavone and mixing 6-methylflavone with TAF. Bitterness was completely and reliably blocked in two of these subjects. These data demonstrate that a combined approach of human taste cell culture–based screening, receptor-specific assays, and human psychophysical testing can successfully discover molecules for blocking perceived bitterness of pharmaceuticals, such as the HIV therapeutic TAF. Our hope is to use bitter taste blockers to increase medical compliance with these vital medicines.

**SIGNIFICANCE STATEMENT:**

Identification of a small molecule that inhibits bitter taste from tenofovir alafenamide may increase the compliance in treating children with human immunodeficiency virus infections.

## Introduction

Bitter taste evolved to indicate when orally sampled substances contain chemicals that we may wish to avoid. Although we may learn to enjoy low level bitterness in certain foods, such as coffee and beer, high intensity bitterness is universally aversive and can induce nausea (Peyrot des Gachons et al., 2011). Common plant alkaloids are often bitter tasting and at high levels can be toxic or even fatal. At low levels, however, many of these bitter stimuli have medicinal properties, for example, the cardiac glycosides. Similarly, the majority of human-made active pharmaceutical ingredients (APIs) used to treat diseases also taste bitter. The bitterness of APIs leads to compliance problems for oral intake of such medicines. For instance, the pediatric population is often extremely sensitive to the adverse taste profile of medicines, and one-third of children with chronic illnesses refuse to take medicines due to strong bitterness or other “bad” sensations elicited by the APIs (Venables et al., 2015).

Since children have difficulty swallowing capsules, liquid formulations expose the bitterness of APIs as consequence. As an example of the life-threatening consequences of bitter-tasting medicine, children with human immunodeficiency virus (HIV) infections must consistently take their antiretroviral medicines; otherwise, they provide opportunity for the virus, which can be fatal, to mutate and become more difficult to treat (Holkmann Olsen et al., 2007; Nachega et al., 2011). Bitterness of the medications is a root cause of “on again/off again” medical compliance in treating children with HIV infections. Parents often resort to restraining and physically forcing their children to take the medicine (Hammami et al., 2004). In some instances, the urgent need for consistent medical compliance is so vital that gastric procedures are undertaken to enable medicines to be placed directly in the stomach via percutaneous endoscopic gastrostomy (Shingadia et al., 2000). A general practice to encourage children to take their bitter-tasting medicines is to add flavoring or sweetening compounds. This strategy, however, has limitations and does not always “mask” bitterness adequately. Therefore, more effective approaches to block bitter taste and increase medical compliance are desperately needed for the ever-evolving bitter-tasting medicines being developed.

Bitterness is the most complicated taste quality from a stimulus-receptor perspective. Bitter taste is mediated by at least 25 TAS2R bitter taste receptors (Bachmanov and Beauchamp, 2007). These G protein–coupled receptors are expressed in taste receptor cells in the oral cavity and signal bitter-tasting stimuli upon entry into the mouth (Bachmanov and Beauchamp, 2007). Targeting these receptors to develop novel bitter taste blockers is of increasing interest both academically and industrially (Adler et al., 2000; Chandrashekar et al., 2000; Matsunami et al., 2000). A few blockers for specific bitter receptors have been reported (Slack et al., 2010; Greene et al., 2011; Pydi et al., 2014; Roland et al., 2014; Zhang et al., 2019). Unfortunately, the efficacy of these blockers to decrease bitter taste perception is limited. This may be partly due to the complicated interactions of bitter-taste stimuli and the TAS2Rs. A single bitter-tasting compound can activate one or several TAS2Rs (Meyerhof et al., 2010). Conversely, the receptive field of a TAS2R may be narrowly or very broadly tuned to many compounds (Meyerhof et al., 2010). Moreover, there are individual polymorphic differences in bitter taste receptor genes and the receptors for which they code; a prime example is the well characterized bitter-taste receptor TAS2R38 (Kim et al., 2003; Bufe et al., 2005). Individuals who carry two copies of the “sensitive” haplotype PAV (proline, alanine, valine at positions 49, 262, and 296, respectively) can perceive phenylthiocarbamide and 6-n-propylthiouracil as strongly bitter. By contrast, individuals who carry two copies of the “insensitive” haplotype AVI (alanine, valine, isoleucine at positions 49, 262, and 296, respectively) typically do not perceive phenylthiocarbamide and 6-n-propylthiouracil as strongly bitter, except at very high concentrations (Kim et al., 2003; Bufe et al., 2005).

Given the complexity of the interaction among the type 2 taste receptors (TAS2Rs) and bitter taste stimuli, such as APIs, we reasoned that characterization of the interaction of a target API with all 25 bitter receptors would be necessary to develop specific bitter taste blockers. Additionally, using human taste bud epithelium-derived cell (hTBEC) cultures that more closely resemble native *in situ* human taste cell physiology would provide an ideal platform for screening chemical libraries for bitter taste modulators of the API (Ozdener et al., 2011).

In the present work, we employed the strategy of combining 1) human taste bud cell culture–based screening, 2) bitter taste receptor assay reporting, and 3) human taste psychophysical testing to identify taste blockers of an intensely bitter-tasting anti-HIV compound, tenofovir alafenamide (TAF) (Ray et al., 2016). Our aim is to reduce the aversiveness of the medicine for children and to increase compliance with their lifesaving treatment. Consequently, we identified TAS2R39 as one of the critical receptors for TAF and showed that the terpenoid 6-methylflavone blocks TAS2R39 responses to TAF at both the cellular and the TAS2R receptor level. We further determined that 6-methylflavone reliably and nearly completely blocked TAF-induced bitterness in a subset of the human subjects we tested.

## Materials and Methods

### hTBEC Platform-Driven Screening of Phytochemical Small Molecules.

All research was conducted according to the principles expressed in the Declaration of Helsinki and approved by an Institutional Review Board at the University of Pennsylvania (no. 821870). Subjects provided written, informed consent on forms approved by the Institutional Review Board prior to human taste bud tissue collection.

The growth and assay of cultured hTBECs was optimized further from the prior foundational work that invented these cultures (Ozdener et al., 2011). hTBECs were grown in a modified BronchiaLife medium (Lifeline Cell Technology, Frederick, MD) with its additives kit (exact concentrations not disclosed by Lifeline), 2.5% FBS, and standard antibiotics mixed 1:1 with Advanced MEM medium (Fisher Scientific/Invitrogen/Gibco-BRL) supplemented with standard antibiotics and standard l-glutamine supplement concentration. To fortify hTBEC attachment in standard culture and in 384-well plate–based medium-throughput screening assays, an extracellular matrix protein coating solution of fibronectin (1 µg/ml final concentration) and gelatin (0.02% final concentration) was also employed. This specialty medium and extracellular matrix coating solution improved the growth and expansion of hTBECs and enhanced their utility in applied science experimentation. These primary cells were used between passages 3 and 9. The average lifetime of an hTBEC primary culture in our hands is 10–12 passages. Cells were seeded at 2500 cells per well in a 384-well plate employed for this primary hTBEC culture. Bitter-responsive hTBEC cultures were identified in foundational work prior to this project. Detection of the principal neurotransmitter ATP secreted by human taste bud cells was assayed by the use of extracellular luciferase and luciferin (ENLITEN kit; Promega, Madison, WI). After stimulation with TAF (300 µM) with and without the addition of small test molecules as potential bitter blockers (10 μM test concentration) in a 45-minute incubation and in an assay buffer that inhibited ecto-NTPases and ecto-NDPases, supernatants were mixed with ENLITEN reagents, and bioluminescence was measured in a BioTek Synergy light reader. A 384-well plate assay design was implemented throughout the assays. CellTiterGLO (version 2.0) was employed to determine if any putative bitter blockers were cytotoxic (which would lower the signals in the assays falsely), and cell-free measurements of putative bitter blockers with ENLITEN reagent were performed to determine if any putative bitter blockers were luciferase enzyme inhibitors. A single concentration validation of the 10 μM test concentration was repeated for every putative bitter blocker to further validate a putative bitter blocker. As a final step, multiple concentration-response curve (CRC)–based validation assays were performed to demonstrate that each putative bitter blocker had a CRC relationship in blocking the stimulation of the hTBEC platform by TAF. This final step provided potency (estimated IC_50_) and efficacy (% inhibition of TAF stimulation) metrics for each putative bitter blocker. With a subset of putative bitter blockers, a Fluo-8/AM–based real-time cell calcium assay was performed as another evidence step for validation by blocking the TAF-elicited cell calcium signals mediated by key TAS2R receptors. Overall, from 2580 phytochemical small molecules screened (as well as a small collection of botanical extracts, all procured from TimTec, Newark, DE), 30 putative hit bitter blockers (25 small molecules and five botanical extracts) were identified, a subset of which were also tested with receptor-based assays and evaluated in human sensory testing as subsequent validation steps.

### Receptor-Based Assays Using Heterologous HEK293 Cells.

Human TAS2R constructs were prepared as described previously (Wang et al., 2019). Functional assays of human TAS2Rs were performed as described previously (Wang et al., 2019). Briefly, human embryonic kidney 293 (PEAKrapid, CRL-2828; American Type Culture Collection, Manassas, VA) cells were seeded on 96-well plates at a density of 25,000 cells per well and cultured in Opti-MEM medium with 4% fetal bovine serum. Next day, cells were then transiently transfected with a TAS2R construct (0.1 µg/well) along with a G protein G*α*16-gust44 (0.1 µg/well) construct by Lipofectamine 2000 (0.5 µl/well). For controls, only G*α*16-gust44 was used (mock transfection). About 20–24 hours after transfection, cells were washed with 10 mM HEPES-supplemented Hanks’ balanced salt solution and then loaded with Fluo-4 in the dark for 1 hour. After incubation, cells were washed two more times with 10 mM HEPES-supplemented Hanks’ balanced salt solution, incubated in the dark for another 30 minutes, and then washed with assay solution once more before running the assay using a FlexStation III reader. Relative fluorescence units (excitation at 494 nm, emission at 516 nm, and auto cutoff at 515 nm) were read every 2 seconds for 2 minutes. Calcium mobilization traces were recorded.

Changes in fluorescence were calculated as the peak fluorescence minus baseline fluorescence (Lei et al., 2015). The calcium mobilization was quantified as the percentage of change relative to baseline. Each data point for bar graphs and concentration-dependent responses was averaged from triplicates (mean ± S.D.). Calcium mobilization traces and bar graphs along with concentration-dependent plots were all generated by GraphPad Prism 5. Analysis of variance with Dunnett’s multiple comparisons test was used for statistical analysis.

### Human Sensory Testing Methods.

All research was conducted according to the principles expressed in the Declaration of Helsinki and approved by an Institutional Review Board at the University of Pennsylvania (authorization no. 701334.) Subjects provided written, informed consent on forms approved by the Institutional Review Board prior to sensory taste testing.

Sixteen healthy adults (11 female, five male; mean age = 42 years, S.D. = 15) participated. Subjects were recruited from among employees of the Monell Chemical Senses Center and the surrounding community and paid for their time.

To determine if 6-methylflavone reduces the bitterness of TAF, subjects rated the perceived bitterness of TAF with and without 6-methylflavone in two separate sessions each with a replicate. The sessions were spaced at least 1 hour apart to prevent carry-over effects from the long-lingering sensations of the test solutions.

All solutions were prepared with 1% Tween 80 (Fisher Scientific), 5% of 75.5% Everclear Grain Alcohol (Local Fine Wine and Spirits Store) and filtered water (Milli-Q Water Purification System), as 6-methylflavone is not water soluble. The TAF solution was prepared by first dissolving TAF (Nanjing Bilatchem Industrial Co., Ltd.) and Tween 80 in filtered water and then adding Everclear Grain Alcohol last. The concentration of TAF was 0.5 mg/ml, which is 0.84 mM TAF (within the range that was tested on cells). The 6-methylflavone prerinse solution was prepared by first dissolving Tween 80 in filtered water and adding Everclear Grain Alcohol with 6-methylflavone (Fisher Scientific) last. The admixture of TAF and 6-methylflavone was prepared by first dissolving TAF with Tween 80 in filtered water and adding Everclear Grain Alcohol with 6-methylflavone last. The concentration of 6-methylflavone in the prerinse and admixture was 1 mM. Solutions were prepared the day of testing and stored at room temperature (about 23°C).

Each subject was instructed to refrain from smoking, eating, chewing gum, and drinking anything, except water, for 1 hour before participation in each session. In the condition with 6-methylflavone, subjects first prerinsed with it four times and expectorated. Then they placed 10 ml of the solution with TAF and 6-methylflavone in their mouth for 5 seconds and expectorated. Immediately after expectorating they rated perceived bitterness on a labeled magnitude scale (Green et al., 1996). In the condition without 6-methylflavone, subjects prerinsed with filtered water (Milli-Q Water Purification System) four times and then placed 10 ml of the TAF solution in their mouths for 5 seconds and expectorated and then immediately rated bitterness. Subjects completed each condition twice while wearing nose clips. The use of this method was due to the unknown kinetic behavior of 6-methylflavone in the oral cavity. Using prerinse and admixture would largely, if not completely, eliminate the potential compounding kinetic effects of 6-methylflavone on suppression of TAF bitterness. Future experiments with simplified dosing regimens are warranted for subjects who show sensitivity to 6-methylflavone to determine what would be the most simple and effective dosing scheme for blocking TAF bitterness using 6-methylflavone.

To control for unspecified taste impairment, we also tested whether 6-methylflavone reduced the bitterness of ferroquine, an effective antimalarial drug. Nine subjects from the original 16 (four female, five male; mean age = 42 years, S.D. = 12.5) participated. The concentration of 6-methylflavone was 1 mM, the same as for the TAF experiment. Ferroquine was dosed at 1200 mg/200 ml. 6-methylflavone was dissolved in 5% of 75.5% Everclear Grain Alcohol (Local Fine Wine and Spirits Store) and filtered water (Milli-Q Water Purification System), as 6-methylflavone is not water soluble. In a separate beaker ferroquine and 1% Tween 80 (Fisher Scientific) were dissolved in 1% hydrochloric acid (Sigma-Aldrich) made with filtered water (Milli-Q Water Purification System). Then the 6-methylflavone mixture was slowly added to the ferroquine solution. The ferroquine solution without 6-methylflavone was prepared by dissolving the ferroquine and 1% Tween 80 in the 1% hydrochloric acid made with filtered water and then slowly adding the 5% of 75.5% Everclear Grain Alcohol. The remaining procedures were identical to those used for testing the effectiveness of 6-methylflavone against TAF.

## Results

### Identification of a Small Molecule that Blocks the Response of Cultured Human Taste Cells to TAF.

**Fig. 1:**
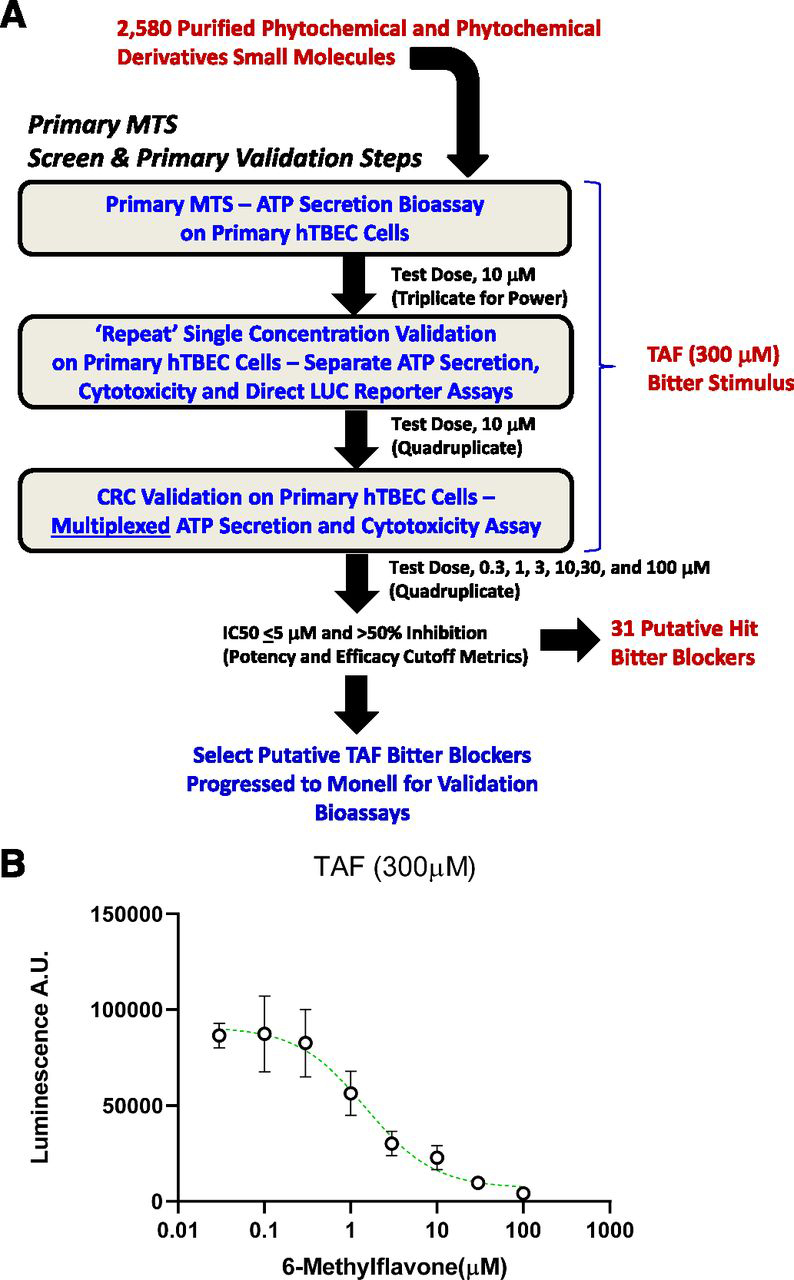
Primary human taste cell–based screening led to identification of 6-methylflavone as a suppressor of TAF-induced taste cell activity. (A) Critical path of bioassays in the MTS design to discover bitter blockers. Cutoff metrics for “hit picking” after the MTS step, validation steps, and counter screen assays are shown. (B) Concentration-response curve for 6-methylflavone in blocking TAF stimulation. TAF was the bitter stimulus in the assay. The IC_50_ was approximately 5 µM for 6-methylflavone, and the efficacy of inhibition for 6-methylflavone was 90% or greater at 30 and/or 100 µM. Each compound was tested in triplicate in the MTS step, and every concentration was tested in quadruplicate in each step thereafter. A.U.: arbitrary units; LUC: luciferase; MTS: medium-throughput screening.

To identify putative blockers for TAF, we first developed and optimized an activity assay using primary hTBEC culture (Ozdener et al., 2011). Aspects of hTBEC culture were also optimized for use as a medium-throughput screening platform (Fig. 1A). In this assay, we measured the secretion of adenosine triphosphate (ATP) from cultured cells in response to taste stimuli. The premise for this assay is that taste bud cells secrete ATP as the principal neurotransmitter in responses to bitter taste stimuli (Finger et al., 2005). As expected, these primary cultured hTBEC cells secrete ATP in response to TAF (Fig. 1B). We screened 2580 natural product–friendly phytochemicals and botanical extracts obtained from TimTec. A number of putative hits emerged from screening, including 6-methylflavone among a small panel of additional flavonoid small molecules, which blocked the response of cultured primary taste cells to TAF in a concentration-dependent manner (Fig. 1B; Supplemental Table 1). Counterscreens were performed to ensure that the putative hit bitter blockers were not cytotoxic and that they did not interfere with light-based reporters and dyes. Blockade of TAF responses was also repeated in three independent experiments, the last of which was a CRC-based assay to ascertain relative potency and efficacy of the putative bitter blocker. Potency of ≤10 μM and efficacy of ≥50% inhibition were cutoff metrics in the hTBEC screen with TAF as a bitter stimulus. A literature survey indicated that structurally similar compounds (e.g., 6-methoxyflavonone) are blockers of TAS2R39.

### TAF Activates a Limited Number of Human Bitter Receptors.

**Fig. 2:**
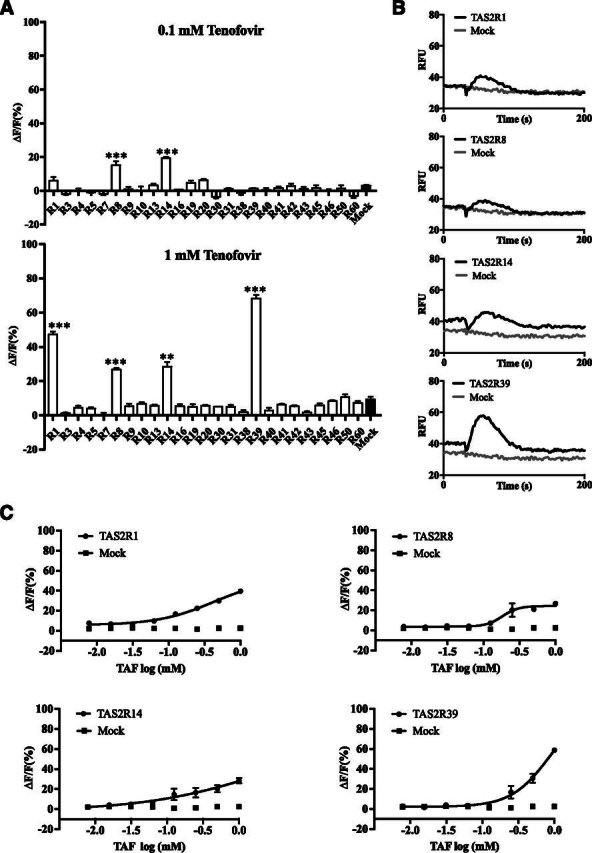
TAF activates a limited number of bitter taste receptors. HEK293 cells transfected with human TAS2Rs, along with G*α*16-gust44, were assayed for their responses to 0.1 mM (A, left) and 1 mM TAF (A, right). Quantitative analysis of responses is presented. Data are percentage change (means ± S.D.) in fluorescence [peak **relative fluorescence units (**RFU) − baseline RFU, denoted Δ*F*] from baseline fluorescence (denoted F) averaged from triplicates (*n* = 3). The experiment was replicated one more time. (B) Representative calcium mobilization traces of TAS2R1-, TAS2R8-, TAS2R14-, and TAS2R39-expressing cells to 1 mM TAF (black traces). Mock-transfected cells (G*α*16-gust44 alone) were used as control (gray trace). (C) TAF activates TAS2R1, TAS2R8, TAS2R14, and TAS2R39 concentration-dependently. Each concentration was tested in triplicate (*n* = 3). Analysis of variance with Dunnett’s multiple comparisons test was used for statistical analysis. ***P < 0.001.

TAF has been reported to be intensely bitter. However, which bitter receptor(s) mediate its bitterness is unknown. To address this question, we used the standard bitter taste receptor assay that couples the receptor activation to calcium mobilization to identify the bitter receptor(s) responsible for the bitter taste of TAF (Chandrashekar et al., 2000; Meyerhof et al., 2010). Each TAS2R was expressed individually in heterologous HEK293 cells, along with a coupling G protein G*α*16-gust44, to examine potential TAF stimulation of each receptor (Ueda et al., 2003; Lei et al., 2015; Wang et al., 2019). For initial characterization, two different concentrations (0.1 and 1 mM) of TAF were tested against all 25 bitter receptors that were transiently transfected. Calcium mobilization was measured using a FlexStation III reader. TAS2R39- and TAS2R1-expressing cells showed robust responses to 1 mM TAF. TAS2R8 and TAS2R14 also showed notable responses to 1 mM TAF (Fig. 2, A and B). At the lower concentration (0.1 mM), cells expressing TAS2R8 and TAS2R14 showed small but significant responses to TAF (Fig. 2A). To further characterize the responses of these four receptors toward TAF, concentration-dependent response curves were obtained for all four receptors. Consistent with the result we obtained above, these four receptors show responses to TAF in a concentration-dependent manner (Fig. 2C). Importantly, TAS2R39 appeared to show the largest response amplitude toward TAF at the highest concentration (1 mM) tested. These data suggest that TAF can activate a limited number of bitter taste receptors.

### 6-Methylflavone Blocks the Response of TAS2R39 to TAF.

**Fig. 3:**
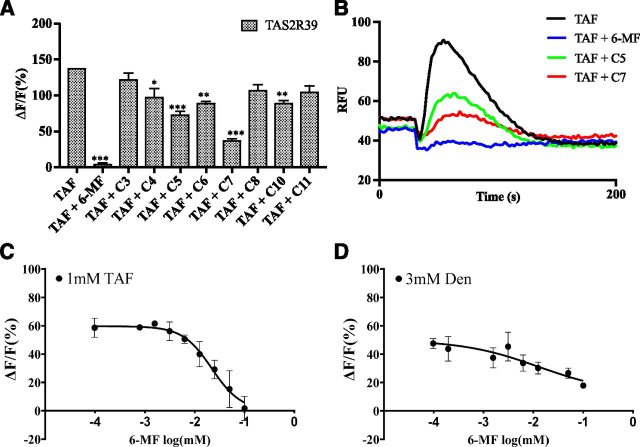
6-methylflavone and its analogs block the responses of TAS2R39 to TAF and denatonium. HEK293 cells were transfected with TAS2R39 and G*α*16-gust44 and assayed for responses to 6-methylflavone, its analogs, and other putative blockers identified in taste cell–based screening (Fig. 1). (A) Bar graph showing the responses of TAS2R39 to TAF (1 mM) in the presence of 6-methylflavone, its analogs, and other putative blockers. 6-methylflavone (0.1 mM) completely blocked the response of TAS2R39 to TAF. Two other analogs (compounds C5 and C7) at 0.1 mM largely blocked the response of TAS2R39 to TAF; three others (compounds C4, C6, and C10) at 0.1 mM partially blocked the response of TAS2R39 to TAF. Data are averaged from triplicates (*n* = 3). The experiment was replicated one more time. Analysis of variance with Dunnett’s multiple comparisons test was used for statistical analysis. ***P < 0.001. **P < 0.01, *P < 0.05. (B) Representative calcium mobilization traces of TAS2R39-expressing cells to TAF, TAF + 6-methylflavone (6-MF), TAF + C5, and TAF + C7. Compounds were added at 30 seconds after the start of the reading. (C) Concentration-response curve showing that 6-methylflavone blocked the responses of TAS2R39 to 1 mM TAF concentration-dependently (IC_50_ = 0.02196 mM) (*n* = 3). (D) 6-methylflavone can block the response of TAS2R39 to the activator 3 mM denatonium (Den) (IC_50_ = 0.01297 mM) (*n* = 3). The experiment was replicated three more times.

Our screening using cultured human taste cells identified 6-methylflavone as a putative bitter blocker. Previous work showed that similar compounds can block the activity of TAS2R39 (Roland et al., 2014). To determine if 6-methylflavone can also block the activity of TAS2R39 toward TAF, HEK293 cells were transiently transfected with TAS2R39 and G*α*16-gust44. We examined the responses of HEK293 cells expressing TAS2R39 and G*α*16-gust44 toward TAF in the presence or absence of 6-methylflavone. In the presence of 0.1 mM 6-methylflavone, the response of TAS2R39 to 1 mM TAF was completely abolished, which suggests that 6-methylflavone is an antagonist of TAS2R39 (Fig. 3, A and B). TAF also activates TAS2R1. To determine if 6-methylflavone may also block the activity of TAS2R1, HEK293 cells expressing TAS2R1 were examined for their responses toward TAF in the presence of 6-methylflavone (Supplemental Fig. 1A). In these cells, no apparent differences were found between the responses of cells to TAF in the presence or absence of 6-methylflavone.

To further characterize the effect of 6-methylflavone on TAS2R39, concentration-dependent curves of TAF responses were obtained. As expected, 6-methylflavone blocked the responses of TAS2R39 to TAF in a concentration-dependent manner (Fig. 3C). In contrast, 6-methylflavone had no effects on the TAF responses of TAS2R1, TAS2R28, and TAS2R14 (Supplemental Fig. 1, B–D). Furthermore, we showed that 6-methylflavone can block the response of TAS2R39 to denatonium, a known TAS2R39 agonist (Fig. 3D) (Meyerhof et al., 2010).

### 6-Methylflavone Analogs Block TAS2R39.

In addition to discovery and validation of 6-methylflavone as a TAS2R39 antagonist, we also identified several other flavonoids as putative bitter blockers from the hTBEC screen of phytochemicals and phytochemical derivatives. Out of a panel of eight compounds (C3, C4, C5, C6, C7, C8, C10, C11; Supplemental Table 2) that we tested, we found five compounds (C5, C7, *P* < 0.001; C6, C10, *P* < 0.01; C4, *P* < 0.05) that also showed effectiveness in blocking TAF-elicited TAS2R39 activity (Fig. 3, A and B). None of these compounds has any notable effect on the responses of TAS2R1 to TAF (Supplemental Fig. 1). We show these data to underscore that the flavonoid phytochemical family has promise in the discovery and development of bitter blockers. However, the most effective at present appears to be 6-methylflavone.

### 6-Methylflavone Reduces Bitterness of TAF in a Subset of Human Subjects.

**Fig. 4:**
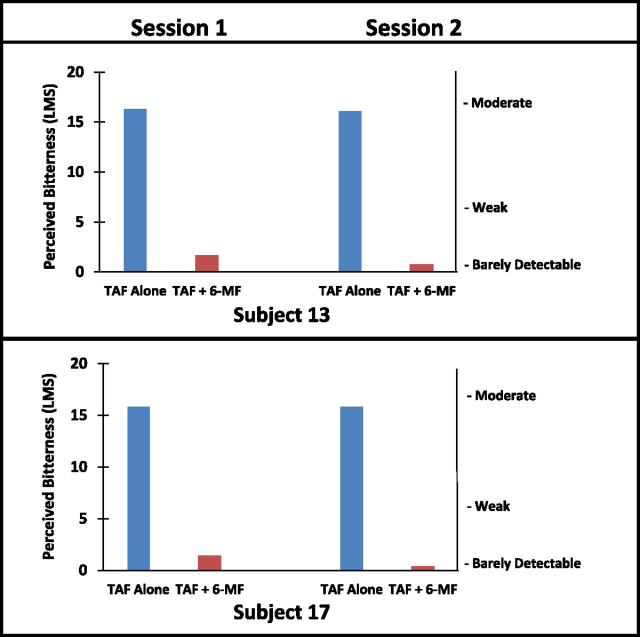
Perceived bitterness of TAF with and without 1 mM 6-methylflavone (6-MF) as prerinse and admixture in two subjects. The top two graphs are ratings by one subject. The bottom two graphs are ratings by another individual subject. The graphs on the left are Session 1, and the graphs on the right are Session 2. In each graph the bar on the left (blue) is perceived bitterness of TAF, whereas the bar on the right (orange) is perceived bitterness of TAF with a 6-methylflavone prerinse and admixture with TAF. TAF was dosed at 0.5 mg/ml. 6-methylflavone was dosed at 1 mM. The solution volume put into the mouth was 10 ml. The bitterness of TAF treated with 6-methylflavone (orange) was nearly completely blocked in these two subjects. These two subjects were repeatedly tested with 6-methylflavone, four repetitions for one subject and five repetitions for the other, to establish the reliability of the observations. The result was very reliable for these two subjects: Pearson *r* = 0.87; *P* < 0.005. LMS: Labeled Magnitude Scale.

**Fig. 5:**
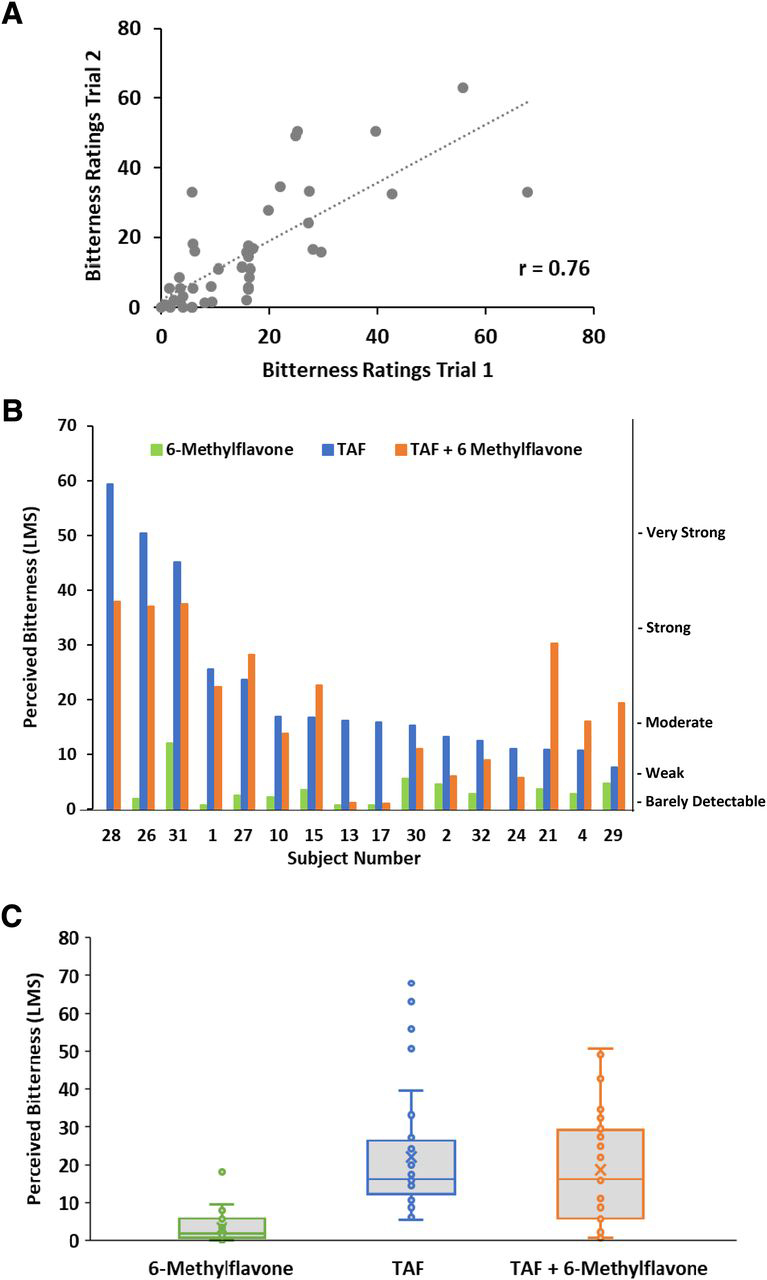
Perceived bitterness of 6-methylflavone, TAF, and TAF with 6-methylflavone as prerinse and admixture in all subjects. (A) The graph shows the test-retest reliability of the subjects’ ratings of perceived bitterness for three conditions: 6-methylflavone, TAF, and TAF with 6-methylflavone as prerinse and admixture. The *x*-axis shows ratings for Session 1, whereas the *y*-axis shows ratings for Session 2. Pearson correlation coefficient = 0.76, *P* < 0.001, indicating consistency and reliability in the subjects’ bitterness ratings across the sessions. (B) The graph shows each subjects’ ratings of perceived bitterness of 6-methylflavone alone (green bar), TAF alone (blue bar), and TAF with 6-methylflavone as prerinse and admixture (orange bar) for all 16 subjects averaged over two replications. TAF was dosed at 0.5 mg/ml, and 6-methylflavone was dosed at 1 mM. The solution volume placed in the mouth was 10 ml. The subjects are sorted from highest to lowest rating of perceived bitterness of TAF. These data highlight the individual differences in bitter taste perception of TAF and bitter taste suppression of TAF with 6-methylflavone. (C) The graph shows subjects’ ratings of perceived bitterness for three conditions: 6-methylflavone, TAF, and TAF with 6-methylflavone as prerinse and admixture. Each condition was tested twice. TAF was dosed at 0.5 mg/ml, and 6-methylflavone was dosed at 1 mM. The solution volume placed in the mouth was 10 ml. The bitterness intensity of TAF is modestly reduced with 6-methylflavone, albeit without statistical significance; repeated measures analysis of variance, *F* (1, 60) = 0.71; *P* > 0.05. LMS: Labeled Magnitude Scale.

To determine if 6-methylflavone reduces the bitterness of TAF, 16 subjects rated the perceived bitterness of TAF with and without 6-methylflavone. On average, 6-methylflavone itself is between “barely detectable” and “weak” in taste at the concentration employed, as tested in these 16 subjects [Fig. 5B (green bars) and Fig. 5C (first bar)]; this was not true for some of the other putative flavonoid bitter blockers that emerged from the screen (data not shown). For 2 of the 16 subjects, the perceived bitterness of TAF was nearly completely and reliably blocked by 6-methylflavone (Fig. 4). These two subjects were repeatedly tested with 6-methylflavone, four repetitions for one subject and five repetitions for the other, to establish the reliability of the observations. In each test the bitterness of TAF was blocked for them to a very large degree. Furthermore, six other subjects had their perceived bitterness of TAF suppressed in both replications, whereas the remaining eight subjects showed either no change or increases in bitterness after prerinsing and presentation of TAF with 6-methylflavone. The test-retest reliability and consistency within the subjects across the two sessions was high (Fig. 5A). Averaged across all subjects tested, 6-methylflavone was only modestly and nonsignificantly effective at reducing bitterness of TAF (Fig. 5, B and C). The reason for the difference in the bitterness-blocking efficacy of 6-methylflavone on TAF among different subjects is currently unclear. However, individual differences in the expression of the appropriate complement of bitter taste receptors that respond to TAF is one explanation. Finally, the effect of 6-methylflavone on TAF shows specificity, as 6-methylflavone did not strongly suppress the bitterness of the antimalarial drug ferroquine in any subjects tested (Supplemental Fig. 2).

## Discussion

In this study, we combined human taste bud epithelium-derived cell–based assays (hTBEC) with bitter receptor-specific cell assays and human taste testing to screen for bitter blockers that can suppress bitterness associated with anti-HIV drug tenofovir alafenamide or TAF. Children with HIV infections must consistently take their antiretroviral medicines; otherwise, they provide opportunity for the virus, which is fatal if not successfully treated, to mutate and become much more difficult to treat. Since children have difficulty swallowing capsules, bitterness of liquid formulations of APIs is a root cause of partial medical compliance in children. In some instances, the need for consistent medical compliance is so vital that surgical procedures are undertaken to enable medicines to be placed directly in the stomach, thus bypassing the bitter taste system.

Our TAF-inhibition screen using native hTBEC culture platforms led to the discovery of 6-methylflavone, which blocks the activation of cultured human taste cells’ response to TAF. Subsequent receptor-based activity assays in heterologous human cells demonstrated that TAF activated a limited number of bitter taste receptors, including TAS2R39, TAS2R1, TAS2R8, and TAS2R14. Using the same heterologous expression assay, we demonstrated that 6-methylflavone inhibits the activity of TAS2R39 to TAF but not of TAS2R1, which may provide a mechanistic explanation of why 6-methylflavone can block the responses of cultured taste cells to TAF. To translate the results from *in vitro* screening into human perception effects, we showed that 6-methylflavone reliably and significantly suppressed the bitterness associated with TAF almost completely in 2 of 16 subjects, partially in six subjects, but not at all in the remaining eight subjects tested. Together, we demonstrated that combining cultured human taste cell–based assays, bitter receptor–based assays, and human taste psychophysical testing provides an efficient and effective way to discover, validate, and develop bitter blockers for subsets of human participants. Clearly, additional blockers will be needed to improve efficacy across a larger proportion of the population.

### Primary Human Taste Cell Culture as a Screening Platform.

The fact that there are 25 human bitter receptors presents some challenges for identification of blockers using high-throughput screening. Screening 25 receptors individually with all bitter APIs of interest and all potential inhibitors requires large-scale resources and efforts. Nevertheless, this approach has been pursued in the biotech industry, albeit in heterologous expression systems (Karanewsky et al., 2016). To circumvent this bottleneck, we used human taste bud cell cultures as a proxy and platform for native human taste cells employing medium-throughput screening of bitter blockers. This screen led to the discovery of several compounds that block activation by TAF and the activation by other bitter-tasting compounds. Despite our success in identification of blockers using primary human taste cell culture, we acknowledge that this approach has limitations. These limitations include cultured cells 1) not representing native taste cells precisely, 2) not yet fully reflecting individual human differences in bitter taste, 3) not targeting specific receptors since native cells tend to express multiple TAS2Rs per cell, and 4) not delineating whether inhibition is based on TAS2Rs or downstream signaling elements. Therefore, to address limitations (3) and (4), we combined initial human taste cell–based screening with receptor-based assays in heterologous human cells for identification of compounds that specifically target human TAS2Rs for further evaluation. And to address limitations (1) and (2), we validated 6-methylflavone as a bitter inhibitor of TAF in human psychophysical tests with a small population sample.

### Receptor-Based Assays and Validation in Human Psychophysical Taste Tests.

Using receptor-based heterologous expression assays, we showed that TAF can activate human TAS2R39 and human TAS2R1 and, to a lesser extent, human TAS2R8 and human TAS2R14. Among these receptors, TAS2R14 when expressed in HEK cells has shown broad tuning; it responds to multiple structurally diverse compounds (Meyerhof et al., 2010; Karaman et al., 2016). The other human TAS2Rs are more narrowly tuned (Slack et al., 2010). Our *in vitro* data suggest that TAS2R39, TAS2R1, and TAS2R8 may be critical receptors for transducing TAF bitterness. We further show that 6-methylflavone was an effective blocker of TAS2R39. Blocking one TAF receptor (TAS2R39), however, may not be sufficient to suppress bitter taste across a large heterogeneous human population (Meyerhof et al., 2010).

In addition to two subjects who reliably and significantly showed almost complete suppression of TAF bitterness by 6-methylflavone, six other subjects also reliably reported reduction of TAF bitterness in the presence of 6-methylflavone. 6-methylflavone was not, however, effective at blocking TAF bitterness for all subjects. TAF was used at approximately 1 mM, a standard patient formulation, which is within the same range that was blocked in cellular assays by 6-methylflavone. Clearly, sensitivity to 6-methylflavone as an inhibitor of TAF is a physiologic and perceptual trait for some but not all people. These individual differences in response to pharmacological agents comprise the basis for individualized (personal) medicine. For example, humans with red hair can carry mutations of the melanocortin-1 receptor that predisposes them to be overly sensitive to morphine treatments (Mogil et al., 2005). Similarly, we believe that sensitivity to 6-methylflavone is a multigenic individual trait, based on several TAS2R responses to treatment. For some subjects, 6-methylflavone significantly and almost completely reduces bitterness; in others it suppresses bitterness moderately, and in still others it makes bitterness stronger.

How will we achieve the suppression of bitterness of TAF for all subjects? One possible explanation for the heterogeneity of inhibition is that the bitterness of TAF is mediated by other receptors, such as TAS2R1 or TAS2R8 (see Fig. 2). Consequently, we believe that the identification of antagonists for these other bitter receptors using our tripartite approach would generate a cocktail of inhibitors that when administered would have potent bitterness inhibitory effects for the majority of subjects. For example, in the case of TAF, we could test whether a cocktail containing 6-methylflavone plus antagonists for TAS2R1 and TAS2R8 would decrease the medicine’s bitterness for the majority of patients. If so, we would be able to increase compliance in taking medicines and ultimately be more successful in treating diseases.

In summary, we provide evidence that a tripartite system—based upon 1) human taste bud cell–based bitter antagonist discovery screen combined with 2) individual TAS2R receptor–based deorphanization and inhibition assays and 3) human psychophysical testing—is an efficient and effective critical path of discovery and validation of bitter taste inhibitors. Here we identified the terpenoid 6-methylflavone as a blocker of the bitter taste of TAF, an antiretroviral, anti-HIV/AIDS medication. These results hold promise for aiding with medical compliance in children who are unable to tolerate the aversiveness of their lifesaving medicines.
