# Supplementary material for: Inhibition of Bitter Taste from Oral Tenofovir Alafenamide
Source: Mol Pharmacol. 2021 May 1;99(5):319–27. doi: 10.1124/molpharm.120.000071 (PMC12164691; doi:10.1124/molpharm.120.000071)
Supplement: Supplementary file 1 [file mmc1-mol71r2_supp.pdf]

Supplementary information

Title: Inhibition of bitter taste from oral Tenofovir Alafenamide (TAF)

Erik Schwiebert[1*], Yi Wang[2, 4*], Ranhui Xi[2], Katarzyna Choma[2], John Streiff[1], Linda J. Flammer[2], Natasha Rivers[2], Mehmet Hakan Ozdener[2], Robert F. Margolskee[2], Carol M. Christensen[2], Nancy E. Rawson[2], Peihua Jiang[2], Paul A. S. Breslin[2,3]

1 Discovery Biomed, Birmingham, AL 35424
2 Monell Chemical Senses Center, Philadelphia, PA 19104
3 Department of Nutritional Sciences, Rutgers University, New Brunswick, NJ 08901
4 Current address: Department of Radiology, Zhongnan Hospital of Wuhan University, Wuhan, China，430071

Manuscript number: MOLPHARM-AR-2020-000071

Flavonoid Compound	Estimated IC50 from CRC	% Inhibition at 30 µM	% Inhibition at 100 µM
6-methylflavone	4.9 µM	85 + 2% on TAF Stimulus	>95% on TAF Stimulus
ST069360	6.9 µM	86 + 3% on TAF Stimulus	>95% on TAF Stimulus
ST098995	4.0 µM	81 + 3% on TAF Stimulus	85 + 2% on TAF Stimulus

Table S1: Estimated IC50s from CRC of 6-methylflavone and its analogs.

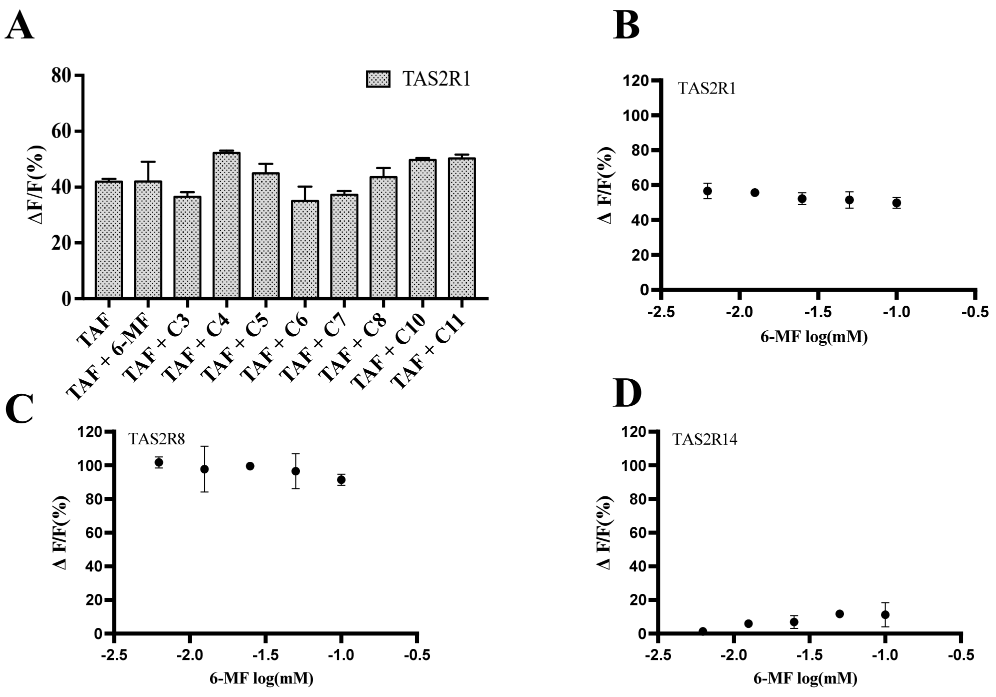

Fig. S1. 6-methylflavone do not block the responses of TAS2R1, TAS2R8, TAS2R14 to TAF and its analogs do not block the responses of TAS2R1 to TAF. A) HEK293 cells were transfected with TAS2R1, and Gα16-gust44 and assayed for responses to 6-methylflavone, its analogs, and other putative blockers identified in taste cell-based screening . Bar graph showing the responses of TAS2R1 to TAF (1 mM) in the presence of 6-methylflavone, its analogs, and other putative blockers (0.1 mM). None of them blocks the response of TAS2R1 to TAF. B-D) HEK293 cells were transfected with TAS2R1,TAS2R8, TAS2R14 and Gα16-gust44 and assayed for responses to 6-methylflavone. Concentration-response curve showing 6-methylflavone at different concentrations does not block the responses of TAS2R1, TAS2R8, TAS2R14 to 1mMTAF. Data are averaged from triplicates. The experiment was replicated one more time.

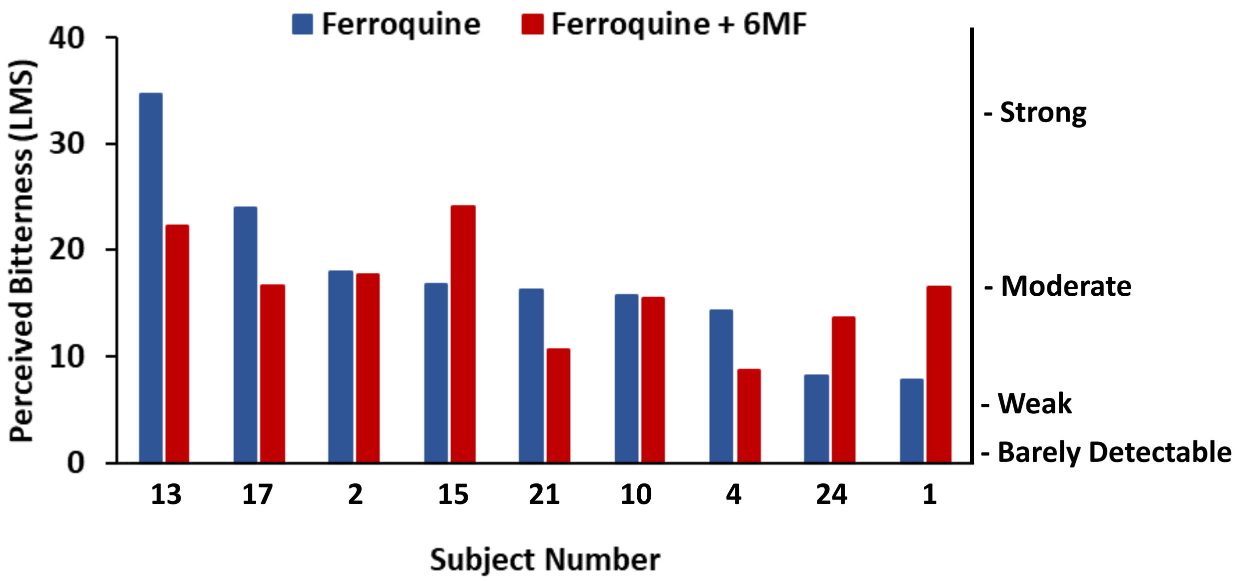

Fig. S2. Perceived bitterness of ferroquine and ferroquine with 6-methylflavone as pre-rinse and admixture.

There are two bars for each of nine subjects in the graph. The dark blue bars show the perceived bitterness of ferroquine (an antimalarial drug) and the red bars show the perceived bitterness of ferroquine with a 6-methylflavone pre-rinse and admixture with ferroquine. The ratings are an average of two replications. Ferroquine was dosed at 1200 mg/200 ml. 6-methylflavone was dosed at 1 mM. The solution volume put into the mouth was 10 ml. The subjects are sorted from highest to lowest rating of perceived bitterness of Ferroquine. There was no difference in perceived bitterness between Ferroquine alone and Ferroquine treated with 6-methylflavone, repeated measures analysis of variance with 2 conditions and 2 sessions, $F(1,32) = 0.20$, $p = 0.66$. Unlike with TAF, there were no individuals who were strongly suppressed by 6-methylflavone.

6-MF	ST069348	0.1 mM	6-methylflavone	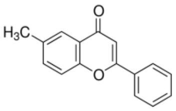
C3:	ST098995	0.1 mM	5,7,4’-trimethoxyflavone	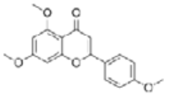
C4:	ST069360	0.1 mM	5-methoxyflavone	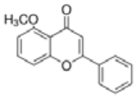
C5:	ST056005	0.1 mM	4’-hydroxyl-6-methoxyflavone	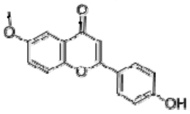
C6:	Pterostilbene	0.1 mM	Stilbene derivative	
C7:	ST043224	0.1 mM	Phytochemical derivative	
C8:	ST044974	0.1 mM	Phytochemical derivative	
C10:	ST025826	0.1 mM	Phytochemical derivative	
C11:	ST104720	0.1 mM	Ononin	

Table S2: The TimTec catalog numbers of 6-methylflavone and its analogs and other phytochemical derivatives tested in receptor-based assays.
